# Fostering Thriving in Healthcare Organizations: An Opportunity to Strengthen the Nursing Professional Workforce

**DOI:** 10.17533/udea.iee.v43n3e02

**Published:** 2025-10-17

**Authors:** Tatiana Osorio Leyton, Tomás Inostroza Ortega, Marcela González Agüero, Camila Lucchini Raies, Lilian Ferrer, Cynthia J. Wensley, Stephen Jacobs

**Affiliations:** 1 Nurse, MSc Nursing, PhD© Nursing. Adjunct Instructor. Email: tposorio@uc.cl https://orcid.org/0000-0003-3980-595X Pontificia Universidad Católica de Chile Chile tposorio@uc.cl; 2 Nurse, MSc Nursing. Assistant Professor. Email: tainostroza@uc.cl. Corresponding author. https://orcid.org/0000-0002-3299-4359 Pontificia Universidad Católica de Chile Chile tainostroza@uc.cl; 3 Nurse. MPH. PhD Medical Anthropology. Associate Professor. Email: mmgonzal@uc.cl. https://orcid.org/0000-0002-0738-4399 Pontificia Universidad Católica de Chile Chile mmgonzal@uc.cl; 4 Nurse-Midwife. PhD. Associate Professor. Email: clucchin@uc.cl. https://orcid.org/0000-0002-3607-6424 Pontificia Universidad Católica de Chile Chile clucchin@uc.cl; 5 Nurse-Midwife. PhD. Senior Lecturer. Email: lferrerl@uc.cl. https://orcid.org/0000-0001-5704-9778 Pontificia Universidad Católica de Chile Chile lferrerl@uc.cl; 6 Nurse. PhD. Senior Lecturer. Email: c.wensley@auckland.ac.nz. https://orcid.org/0000-0002-6224-7514 The University of Auckland New Zealand c.wensley@auckland.ac.nz; 7 Researcher. PhD. Senior Lecturer. Email: s.jacobs@auckland.ac.nz. https://orcid.org/0000-0002-0919-7351 The University of Auckland New Zealand s.jacobs@auckland.ac.nz; 8 Nursing School. Pontificia Universidad Católica de Chile. Santiago de Chile Pontificia Universidad Católica de Chile Nursing School Pontificia Universidad Católica de Chile Chile; 9 Nursing School. The University of Auckland. Auckland, New Zealand The University of Auckland Nursing School The University of Auckland Auckland New Zealand

## Introduction

Nursing professionals represent the largest segment of the global healthcare workforce, playing a vital role in patient care, clinical management, and the ongoing support of healthcare systems.^1^ Nevertheless, despite this relevance, it is estimated that by 2030 the global deficit of nurses will reach 4.5 million,^2^ which represents a critical challenge for the capacity of healthcare systems to respond in a timely, safe, and equitable manner to the needs of populations. In this regard, the need to promote strategies that strengthen human resources in nursing has been widely recognized.^3^ Notwithstanding, many of these strategies have focused primarily on mitigating the effects of unfavorable work and organizational environments on the nursing workforce. These include burnout, psychological stress, moral distress, and work-life imbalance.^4^ While addressing these issues is necessary, this has proven insufficient to reverse the progressive deterioration of the working conditions of nursing staff. Insisting on this approach represents the risk of continuing to only address the symptoms without transforming the structural causes.

Given this scenario, this editorial holds that a paradigm shift is essential that conceives organizations as living and dynamic ecosystems, capable of intentionally managing their work contexts to promote sustainable wellbeing over time. Such requires an organizational culture that assumes that the responsibility for wellbeing is shared between each individual and the institution. Only thus will it be possible to move from reactive responses to preventive strategies and the genuine fostering of well-being, creating environments where nurses not only remain, but can thrive. “Thriving at work” alludes to the capacity of individuals to grow, develop, and feel energized, in contrast to perceptions of stagnation or exhaustion.^5^ The experience of thriving has been related positively with greater job satisfaction, better performance and commitment at work, better subjective perception of health, respectful organizational behaviors, as well as with lower levels of exhaustion, burnout, and intention to rotate or leave the profession.^6^ Moreover, the benefits of thriving are not only restricted to the individual plane; professionals who thrive tend to get involved actively in improving their organizations, contributing ideas, promoting collaboration, and exercising proactive leadership.^6^

Most studies about thriving at work come from Anglo-Saxon contexts, which limits their universal applicability. Hence, addressing this issue in various contexts is key to ensuring that nursing professionals not only remain in their positions but also find spaces to grow and actively contribute to strengthening healthcare systems.

### Socially embedded model of thriving at work

The “Socially Embedded Model of Thriving at Work” proposed by Dr. Gretchen Spreitzer *et al*.,^7^ defines “thriving at work” as a psychological state in which individuals experience both a sense of vitality (having energy) and a sense of learning (acquisition of knowledge) at work.^7^ This model provides a comprehensive understanding of how individuals interact with their organizational environment, and how this interaction can foster (or hinder) their development and wellbeing. According to the socially embedded model of thriving in the workplace, the act of thriving is the result of a dynamic and bidirectional interaction between the employee's personal characteristics and the attributes of the organizational context.^7^^,^^8^ That is, it is not sufficient for people to have motivation or individual capabilities; they must also have an environment that favors the deployment of these potentialities. Due to this, the model highlights the responsibility of organizations to provide work environments that support their workforce, ensuring access to key resources, like, for example, growth and recognition opportunities.^9^ When the work environment offers adequate conditions, such as autonomy in work, a climate of trust, and meaningful relationships between colleagues and managers, people not only manage healthy adaptation to their environment, but also generate new personal resources, strengthen their performance, and consolidate a sense of continuous progress.^7^^,^^8^ Individuals are not expected to thrive in isolation, making this model inherently social in nature. That implies that thriving cannot be experienced without the influence of interactions with the work environment, its characteristics, colleagues, and managers.^7^

### Nursing and thriving at work

Although the model of thriving in the workplace originated over 20 years ago and has gained growing interest in healthcare organizations, the study of thriving among nursing professionals is still in its early stages. Nonetheless, it has been demonstrated that the model is adequate for application in the nursing staff, especially to serve as a framework for managers to encourage their teams to thrive.^10^ From the model’s perspective, the work environment must meet certain contextual conditions that favor the capacity of nursing professionals to thrive. These conditions are determined by the resources available in the organization and by the quality of relationships with managers and colleagues.^(11)^ The literature suggests that social support, effective supervision, and the necessary resources to perform the role safely and competently are key elements to promote thriving in the workplace.^12^ Particularly, work environments focused on caring for people and promoting psychological empowerment are associated positively with a greater experience of thriving among the nursing staff.^13^

Furthermore, nursing professionals are individuals with specific personalities and psychological resources. Research has identified that their psychological capital (advantageous characteristics, like optimism, resilience, and self-efficacy) is favorably associated with their thriving.^(14)^ Thereby, it is key for nursing professionals to have the necessary support to develop and maintain these individual characteristics, given that such will allow them to be more aware of their role within the organization and of their relationships with peers, favoring bonds characterized by mutual attention (or according to the model, developing “heedful relating”).^14^ Likewise, said professionals would tend to adopt behaviors liketask focus and exploration of their own role, which will increase the probability of learning and experiencing high levels of energy and vitality.^8^^,^^15^ It has been proposed that, when the interaction between individual and contextual characteristics is favorable, it is possible to build work environments where people not only thrive, but maintain that experience in a sustained manner over time. This continuity in thriving not only benefits those who experience it, but also has a positive impact on healthcare organizations because it is associated with greater satisfaction with the care provided, reduction of stress symptoms,^16^ and better job performance by the nursing staff.^17^

### Challenges for thriving in nursing in Latin America: urgency of a paradigm shift

The nursing situation in Latin America evidences persistent structural challenges that seriously hinder conditions for thriving in the workplace. In the region, studies on the nursing workforce are still limited, but the available data allows measuring a critical reality that requires urgent attention. Currently, Latin America has approximately 5.6 million nursing professionals.^18^ However, a study in the region of the Americas in 2017 reported a marked heterogeneity in the density of nursing staff, with countries that exceed eight nurses per thousand inhabitants and others that do not reach one.^19^ Beyond the number of professionals, issues have been identified, such as a lack of team cohesion and stressors linked to ineffective leadership and organizational climate,^20^ high burnout levels,^21^ and high rates of work-related fatigue at the regional level due to multiple jobs, lack of sleep, work overload, sustained physical effort, and experiences of suffering arising from the daily clinical practice.^22^

The lack of organizational policies that promote thriving environments seems to be a regional constant, and in the face of this panorama, the need for a paradigm shift becomes evident. It is no longer enough to demand individual resilience or implement fragmented and reactive actions. It is indispensable for healthcare organizations to be recognized as dynamic ecosystems that can be actively configured to foster the thriving of their workers. This implies not only having structural resources but also designing organizational cultures that promote continuous learning, a sense of purpose, and sustained wellbeing. Within this context, the social model of thriving in the workplace offers a powerful tool to rethink the working conditions of the nursing staff in Latin America. Its application will allow guiding organizational strategies towards more humane, adaptive, and healthy work environments, where professionals can not only survive, but thrive.

In response to this challenge, the School of Nursing at Pontificia Universidad Católica in Chile is actively promoting this research agenda in conjunction with an international network, through its participation in the Nurses Thriving at Work Research Collaborative*.*^4^ This collaboration seeks to work with healthcare professionals to strengthen nursing workforce management practices and generate knowledge that fosters healthier and more enriching work environments, where nurses not only remain in their jobs but also have the support they need to thrive. 

In Chile, two research projects are currently being undertaken aimed at strengthening the thriving of nursing professionals in their work environments. The first corresponds to a longitudinal action research study with early-career nurses and nursing leaders. This study is led by the School of Nursing at The University of Auckland, New Zealand. During this year, the first phase has been implemented, under the coordination of the School of Nursing at Pontificia Universidad Católica in Chile, centered on the cultural adaptation of the instruments to measure thriving at work of nursing professionals. The second phase, to be carried out next year, contemplates the participation of nurses in early stages of their career who will be invited to express the aspects they consider most relevant in their work environments and in the development of their careers. Likewise, meetings will be held among them and nursing leaders to co-design more relevant and effective support systems. The second project underway is aimed at generating recommendations that foster work environments that facilitate thriving through career growth opportunities for nurses in the country. This project is led by a professor from Pontificia Universidad Católica in Chile, who is currently pursuing doctoral studies at The University of Auckland. Both initiatives seek to generate contextualized evidence for the Latin American region and promote the implementation of structural changes in healthcare organizations. Moving forward in this direction constitutes a strategic commitment to strengthen the quality, sustainability, and response capacity of healthcare systems in the region.

In conclusion, the need to enrich work environments in healthcare is urgent. Hence, it is time to move towards organizational strategies that promote career growth, recognition, and well-being of nursing professionals. In this sense, the “Socially Embedded Model of Thriving at Work” represents a promising path to transform work environments in a sustainable and contextualized manner. Exploring how to translate this model into concrete, culturally sensitive, and feasible interventions in resource-limited contexts represents the next big challenge for nursing research and management in the region.
